# Identification of hospitalized mortality of patients with COVID-19 by machine learning models based on blood inflammatory cytokines

**DOI:** 10.3389/fpubh.2022.1001340

**Published:** 2022-11-17

**Authors:** Zhixiang Yu, Xiayin Li, Jin Zhao, Shiren Sun

**Affiliations:** ^1^Department of Nephrology, Xijing Hospital, Fourth Military Medical University, Xi'an, China; ^2^First Unit, Third Branch of Fangcang Shelter Hospital of National Exhibition and Convention Center, Shanghai, China

**Keywords:** COVID-19, outcome, inflammatory cytokines, machine learning, prognostic models

## Abstract

Coronavirus disease 2019 (COVID-19) spread worldwide and presented a significant threat to people's health. Inappropriate disease assessment and treatment strategies bring a heavy burden on healthcare systems. Our study aimed to construct predictive models to assess patients with COVID-19 who may have poor prognoses early and accurately. This research performed a retrospective analysis on two cohorts of patients with COVID-19. Data from the Barcelona cohort were used as the training set, and data from the Rotterdam cohort were used as the validation set. Cox regression, logistic regression, and different machine learning methods including random forest (RF), support vector machine (SVM), and decision tree (DT) were performed to construct COVID-19 death prognostic models. Based on multiple clinical characteristics and blood inflammatory cytokines during the first day of hospitalization for the 138 patients with COVID-19, we constructed various models to predict the in-hospital mortality of patients with COVID-19. All the models showed outstanding performance in identifying high-risk patients with COVID-19. The accuracy of the logistic regression, RF, and DT models is 86.96, 80.43, and 85.51%, respectively. Advanced age and the abnormal expression of some inflammatory cytokines including IFN-α, IL-8, and IL-6 have been proven to be closely associated with the prognosis of patients with COVID-19. The models we developed can assist doctors in developing appropriate COVID-19 treatment strategies, including allocating limited medical resources more rationally and early intervention in high-risk groups.

## Introduction

Syndrome coronavirus-2 (SARS-CoV-2) is the causative agent of coronavirus disease 2019 (COVID-19), which infected more than 180 million people. Compared with a similar acute respiratory syndrome caused by the severe acute respiratory syndrome coronavirus, COVID-19 seems milder but more infectious (1). After being infected with COVID-19, patients' characteristics vary. Some patients became clinically asymptomatic or had mild cases of fever, cough, fatigue, and other symptoms. However, some people became patients with severe COVID-19 and even lost their lives (2–4). Due to inappropriate assessments and treatment, strategies were detrimental to the patient's health and promoted SARS-CoV-2 to become a global societal problem, which has caused over 4 million deaths to date (5). Therefore, identifying patients with poor prognoses as early as possible for necessary interventions is an essential direction for the treatment of COVID-19, which will significantly improve the prognosis of patients and release a tremendous burden on the medical care system.

The immune system and inflammatory syndrome have been proven to play a crucial role in COVID-19 infection (6). Inflammatory cytokines are critical mediators that oversee and regulate immune and inflammatory responses *via* complex networks and serve as biomarkers for many diseases (7). According to previous studies (8–10), inflammatory cytokines were closely related to the progression, complications, and mortality of COVID-19. Universally, these studies paid attention to the relationship between cytokines and disease severity. However, few researchers specifically employed cytokines to construct a model for predicting the prognosis of patients with COVID-19.

Machine learning (ML) algorithms have been widely applied in the medical field, including diagnosing and predicting prognosis. ML models are also used in every aspect of the diagnosis and treatment of COVID-19 due to their fantastic data processing capabilities. Previous ML studies have used multiple indicators, including clinical and blood text indicators, to determine the prognosis of patients with COVID-19. Due to cytokine tests' simplicity, high efficiency, and accuracy, they gradually became an alternative plan for the early prediction of COVID-19 prognosis. Abers et al. (11) fit a Cox proportional hazard to screen the mortality-related inflammatory cytokines. Patterson et al. (12) applied ML methods for the early identification of patients with severe COVID-19 based on cytokines. Mueller et al. (13) classified patients with COVID-19 into different subgroups according to inflammatory cytokines and applied the immunotypes to predict long-term post-COVID-19 complications. However, there are no available models for early prediction of the death of patients with COVID-19 based on blood inflammatory cytokines for clinical work. Constructing a predictive model that can be applied in the clinic seemed urgent. Therefore, in this research, we made use of data collected by Mueller et al. (13), containing 138 inpatients with COVID-19, to construct multiple models based on different algorithms, including logistic regression, random forest (RF), and decision tree (DT) to predict patient deaths.

## Methods

### Data acquisition

In this research, we obtained COVID-19 data from a dataset of Mueller et al. (13) after obtaining author approval. The data were from a finished cohort study, which has been approved by the Ethics Committee for Research with Medicines of Hospital Universitari Vall d'Hebron and Erasmus University Medical Center (13). Therefore, we do not require reapproval from the ethics committee for this study. The dataset contained 138 patients with COVID-19 from two independent cohorts (Rotterdam cohort *n* = 50 and Barcelona cohort *n* = 88). Clinical parameters and laboratory data for each patient during the first 24 h of hospitalization were included in the dataset. Final clinical outcomes were classified into discharge from the hospital and in-hospital death. All inpatients in the study cohort were older than 18 and SARS-CoV-2 positive diagnosed by reverse transcription-polymerase chain reaction (RT-PCR) test and were sampled at hospital entry. Dataset included cytokines measured through the ELLA Simple Plex system and ELISA kits. The COVID-19 antibody concentration in serum was measured through ELISA, and other clinical indicators were obtained by routine tests. Data acquisition is presented in the study by Mueller et al. (13).

### Data preprocessing

First, we binarized the outcome variables into Booleans. We used mean value interpolation to substitute missing values, which was widely used and proved effective for missing values in datasets. Thankfully, no missing values in the COVID-19 patients' cytokines values ensured the model's reliability. We used the Barcelona cohort to construct the model and applied the Rotterdam cohort as an independent external validation set.

Patients' inflammatory cytokine values from the training and validation datasets were loaded into R software (version 4.1.0). R packages “vegan” and “stats” were applied to perform the principal component analysis (PCA) and draw the PCA figure. Besides, we used “ggplot2” for the figure drawing.

Our research selected the following five algorithms: Cox regression, logistic regression, RF, support vector machine (SVM), and decision tree (DT). COVID-19 cases located in different areas were conducted to verify the accuracy of each model to ensure the model's reliability. In the logistic regression model, we input all the inflammatory cytokine values, age, and sex into the logistic regression in the training set. Then, the variables with *p* < 0.1 in the single logistic regression were filtered for the following research. We applied multiple logistic regression (backward: likelihood ratio method) multivariate analysis for the hub variables, and the coefficients derived were used to generate a prognostic model. A prognostic model was constructed based on the logistic regression coefficients. For verification, we generated ROC and calibration curves to calculate the model results for the training set and validation set. The area under the curve (AUC) and 95% confidence interval (CI) were used to verify the model efficiency. Moreover, to obtain a more comprehensive evaluation of the application value of the signature, decision curve analysis (DCA) was performed, demonstrating the net benefit to the patients with COVID-19 after applying the model for prognosis. These steps above were finished using SPSS (version 26.0) and Stata software (version 16). The Cox regression was performed using STATA (version 16), too. The Cox prognostic model was based on the Cox regression coefficients. The ROC and DCA were both drawn to evaluate the model.

In supervised ML, we evaluated the residuals of both methods for the model's accuracy and compared the residuals in our research. After confirming that the RF model is a better method with fewer residuals, we created an RF model to obtain the variables' significance. Artificial neural network (ANN) model training was performed, and the ROCs were used to verify the model efficiency. What is more, we constructed a DT based on all inflammatory cytokines, sex, and age through the R package “rpart” and calculated the accuracy of the DT in the training set and validation set. The RF model was constructed using R packages “randomForest” and “neuralnet.” DT was finished through R package “rpart.” Additionally, the R package “pROC” was applied to perform ROC.

**Figure 1:**
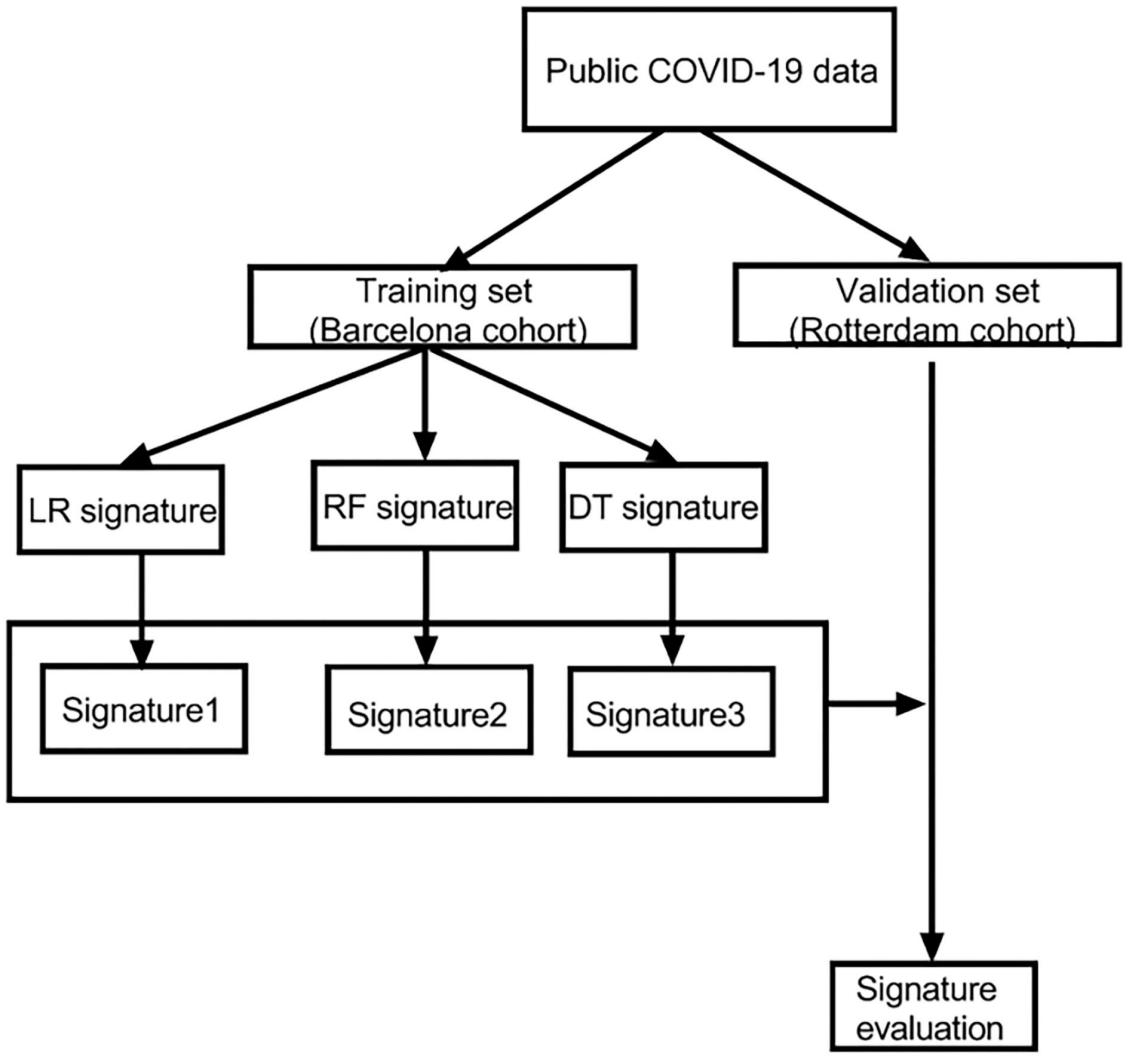
The flowchart of our research. LR, logistic regression; RF, random forest; DT, decision tree.

A flowchart is shown in the Figure 1 to help readers better understand the complete analysis steps.

## Results

### Characteristics of the patients

There are 138 patients with COVID-19 participating in our study cohort. There were 27 (19.6%) deceased cases, containing 18 (13.0%) dead cases in the Barcelona cohort and 9 (7.0%) dead cases in the Rotterdam cohort. In Table 1, we listed the characteristics of each variable in both cohorts. We found no significant differences between the two cohorts, which ensures the feasibility and rationality of applying both datasets to model and validate.

**Table 1:** Characteristics of patients with COVID-19.

**Variables**		**Training cohort and testing cohort**		***P* value**
	**All patients (*n* = 138)**	**Barcelona cohort (*n* = 88)**	**Rotterdam cohort (*n* = 50)**	
Age	62 (54–70)	61 (50–70)	63 (57.25–69)	0.072
Male (n, %)	91 (65.9%)	58 (65.9%)	33 (66.0%)	0.991
**WHO score (Entry)**				0.000
3	38 (27.5%)	34 (38.6%)	4 (8.0%)	
4	60 (43.5%)	31 (35.2%)	29 (58.0%)	
5	20 (14.5%)	12 (13.6%)	8 (16.0%)	
6	12 (8.7%)	10 (11.4%)	2 (4.0%)	
7	8 (5.8%)	1 (1.1%)	7 (14.0%)	
**Laboratory test**
CRP (mg/L)	113 (70, 117.4)	129.5 (76.7,186.8)	97.5 (57.8, 151.4)	0.126
Ferritin (μg/L)	757 (406.7, 1,031.5)	712 (375.8, 954)	882.5 (494.5, 1,131)	0.122
Leukocytes (x10^9^/L)	7.6 (6.1, 9.9)	7.0 (5.1, 10.0)	7.5 (5.9,9,8)	0.338
Neutrophils (x10^9^/L)	6.1 (4.6, 8.0)	5.5 (3.7, 7.7)	5.9 (4.2, 8.0)	0.223
Lymphocytes (x10^9^/L)	1.1 (0.8, 1.4)	1.1 (0.8, 1.5)	1.1 (0.8, 1.4)	0.506
Monocytes (x10^9^/L)	0.5 (0.3, 0.7)	0.4 (0.3, 0.6)	0.5 (0.3, 0.6)	0.164
Thrombocytes (x10^9^/L)	211 (165.5, 288.8)	234 (150.8, 299)	223 (163.5, 292)	0.978
**Outcomes**
LOS	13.5 (4, 30.3)	7 (2, 33.25)	14.5 (7.75, 24.75)	0.108
ICU LOS	0 (0, 16.3)	0 (0, 17)	0 (0, 15)	0.759
Mortality	27	18	9	0.825

BRI, balanced response immunotype; EXI, excessive inflammation immunotype; LAI, low antibody immunotype; CRP, C-reactive protein; LDH, lactate dehydrogenase.

### Building a logistic regression model to classify patients and evaluate the model

We compared the patients with COVID-19 with different outcomes. There existed a significant difference between survival and death cases in age, some antibodies, and cytokines, indicating that these characteristics might play a key role in promoting death (Table 2).

**Table 2:** Characteristics of two cohorts.

**Variables**	**Barcelona cohort (*n =* 88)**			**Rotterdam cohort (*n =* 50)**		
	**Survival (*n* = 70)**	**Mortality (*n* = 18)**	***P* value**	**Survival (*n* = 41)**	**Mortality (*n* = 9)**	***P* value**
Age	57.16 ± 13.8	70.4 ± 10.5	0.000	63.4 ± 10.1	71.8 ± 15.0	0.047
Male (n, %)	49 (70%)	9 (50%)	0.095	27 (65.9%)	6 (66.7%)	1.000
**Laboratory data**
CRP (mg/L)	125.8 (72.7, 183.7)	129.5 (102.0, 249.7)	0.2	101 (69.3, 148)	108 (43.8, 220.8)	0.97
Ferritin (μg/L)	649.5 (324,958.3)	954 (601.8, 954.0)	0.221	882.5 (539.3, 1,108)	856.5 (485.5, 1,177.5)	0.82
Leukocytes (10^9^/L)	7.5 (6.2, 9.6)	8.5 (5.9, 12.4)	0.277	7.2 (5.2, 10.3)	6.4 (4.1, 10.4)	0.419
Neutrophils (10^9^/L)	5.9 (4.7, 7.8)	6.6 (4.4, 9.8)	0.238	6.1 (3.8, 8.3)	4.7 (3.3, 5.6)	0.289
Lymphocytes (10^9^/L)	1.1 (0.9, 1.4)	0.9 (0.8, 1.3)	0.333	1.1 (0.8, 1.5)	0.9 (0.7, 1.3)	0.254
Monocytes (10^9^/L)	0.5 (0.4, 0.7)	0.4 (0.3, 0.8)	0.63	0.4 (0.3, 0.6)	0.3 (0.2, 1.1)	0.361
Thrombocytes (10^9^/L)	211 (166.5, 282)	219 (154.5, 300.5)	0.926	244.8 (178.0, 332.8)	126.5 (85.3, 184.8)	0.005
**Cytokines and anti-body**
anti-N IgM	11 (11, 13.9)	11 (11, 15.4)	0.911	11 (11.0, 23.7)	11 (11.0, 11.0)	0.045
anti-N IgG	11.6 (11, 27.5)	11 (11, 39.8)	0.819	18.6 (11.0, 33.1)	11.0 (11.0, 11.9)	0.014
anti-N IgA	20.7 (11, 61.7)	11 (11,64.1)	0.415	21.0 (11.0, 49.1)	11.0 (11.0, 12.8)	0.011
TGFb1	32,599.5 (23,850, 40,977.8)	31,597.5 (20,318, 44,350)	0.664	35,709 (26,997.5, 48,028.5)	18,182.5 (10,165.3, 30,744.0)	0.003
IL5	0.3 (0.1, 0.5)	0.4 (0.1, 1.4)	0.259	0.7 (0.4, 1.7)	0.9 (0.1, 4.0)	0.544
IFNg	7.2 (2.6, 18.6)	2.3 (1.1, 7.6)	0.014	7.4 (2.8, 19.3)	14.8 (3.4, 35.1)	0.456
IFNa	7.8 (1.5, 27.0)	13.6 (1.5, 75.9)	0.47	5.1 (1.5, 15.5)	22.7 (2.0, 78.8)	0.038
CCL2	541.5 (381, 804.3)	766.5 (552, 1,024.3)	0.019	671.0 (520.5, 1,198.3)	1,285.0 (839.3, 1,971.3)	0.086
IL6	44.8 (25.2, 87.4)	106 (47.6, 148.5)	0.007	38.3 (20.8,62.8)	375.3 (51.3, 1,495.3)	0.003
TNFa	18.8 (14.8, 23.6)	24.0 (14.7, 28.3)	0.289	18.6 (14.4, 22.0)	23.6 (17.8, 28.7)	0.029
IL1b	0.6 (0.2, 1.1)	0.5 (0.3, 1.0)	0.692	0.4 (0.2, 0.6)	0.5 (0.4, 1.0)	0.087
IL8	71.1 (40.7, 136.3)	90.1 (62.2, 316.5)	0.053	44.6 (27.2,80.4)	76.9 (32.8, 160.3)	0.093
IL18	385 (314.5, 512)	495.5 (343.3, 851.5)	0.044	493.5 (411.3, 673.0)	590.5 (396.8, 711.0)	0.544
IL10	13.6 (6.7, 22.1)	21 (15.7, 27.9)	0.02	10.4 (7.0, 17.9)	24.1 (16.0, 32.9)	0.003
IL4	0.3 (0.3, 0.3)	0.3 (0.3, 0.3)	0.576	0.3 (0.3, 0.3)	0.3 (0.3, 0.3)	1
IL2	0.5 (0.5, 0.5)	0.5 (0.5, 0.5)	0.785	0.6 (0.6, 0.6)	1.2 (0.6, 1.9)	0.005
IL12p70	0.5 (0.5, 0.5)	0.5 (0.5, 0.5)	0.83	0.5 (0.5, 0.5)	0.5 (0.5, 0.5)	1
IL17A	1.1 (1.1, 1.1)	1.1 (1.1, 1.1)	0.246	1.1 (1.1, 1.1)	4.1 (2.7, 9.5)	0

All the inflammatory cytokines, sex, and age of the training set were used as input variables to perform single logistic regression. Five variables, including IL8, IL6, IFN-α, and IL17-α, and age with *p* < 0.1 might play a vital role in the COVID-19 patients' classification (Table 3). Multivariate analysis revealed that there were four variables with *p* < 0.05. The logistic regression model was constructed based on these four hub variables (Table 3).

**Table 3:** Logistic regression analysis for mortality of patients in the training set.

**Characteristics**	**Univariate logistic regression**		**Multivariate logistic regression**	
	**OR (95%CI)**	***P* value**	**OR (95%CI)**	***P* value**
Sex	0.739 (0.259, 2.108)	0.571	-	-
Age	1.107 (1.046, 1.173)	0.000	1.140 (1.051, 1.236)	**0.002**
TGFb1	1.000(1.000, 1.000)	0.153	-	-
Il5	1.194 (0.908, 1.569)	0.204	-	-
IFNg	0.959 (0.892, 1.032)	0.261	-	-
IFNa	1.016 (1.002, 1.030)	0.029	1.023 (1.033, 1.044)	**0.026**
CCL2	1.000 (1.000, 1.000)	0.854	-	-
Il6	1.001 (1.000, 1.002)	0.057	1.002 (0.999, 1.004)	0.179
TNFa	1.037 (0.985, 1.092)	0.167	-	-
IL1b	1.484 (0.741, 2.971)	0.265	-	-
IL8	1.004 (1.001, 1.007)	0.022	1.004 (1.001, 1.008)	**0.015**
IL18	1.001 (0.999, 1.002)	0.458	-	-
IL10	1.008 (0.992, 1.024)	0.337	-	-
IL4	1.941 (0.694, 5.429)	0.206	-	-
IL2	4.727 (0.587, 38.075)	0.145	-	-
IL12p70	0.954 (0.57, 1.595)	0.857	-	-
IL17A	1.689 (1.072, 2.662)	0.024	1.989 (1.140, 3.467)	**0.015**

The bold values mean that the P-value < 0.05.

**Figure 2:**
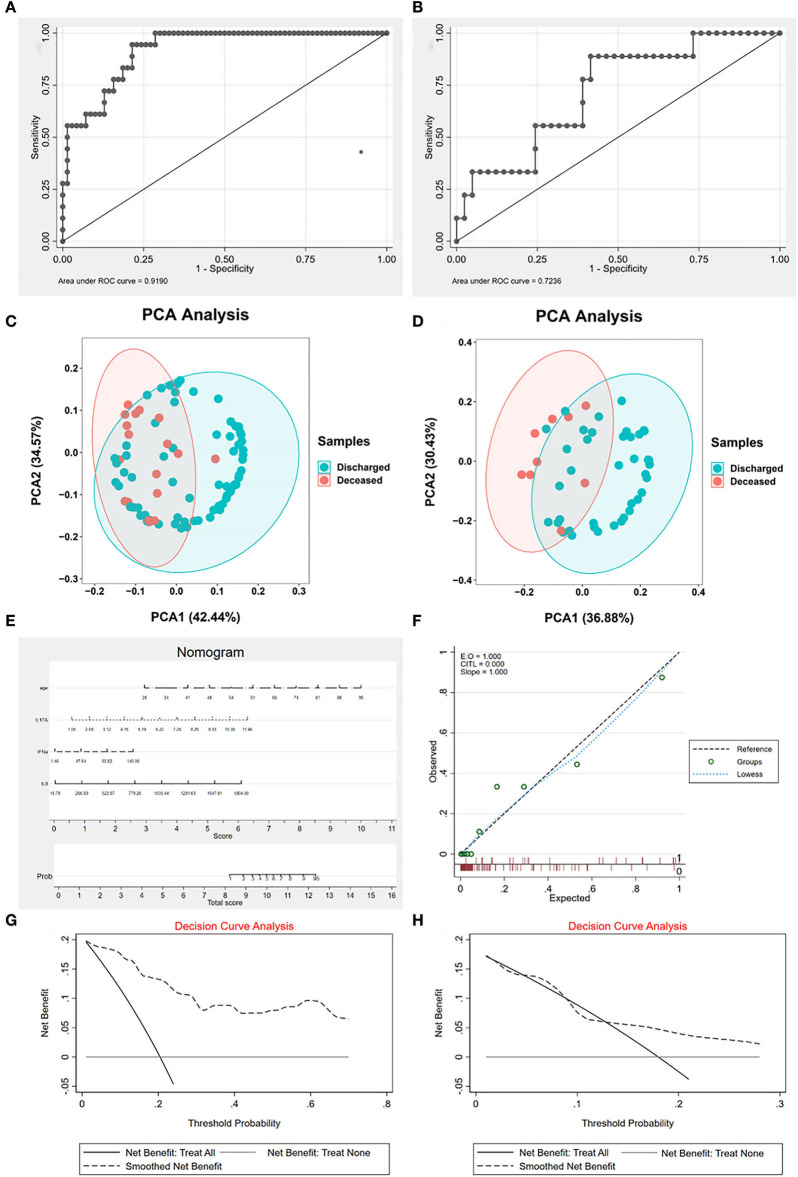
The characteristics of logistic regression (LR) signature. **(A)** The receiver operating characteristic curve (ROC) of signature in the training set. The area under the curve (AUC) is 0.919, indicating that the signature works well in the training set. **(B)** The ROC of signature in the validation set. AUC is 0.7236, indicating that the signature is valuable in the validation set. **(C)** The principal component analysis (PCA) of hub cytokines and age shows that patients with COVID-19 with different outcomes are separate in the training set. **(D)** The PCA of hub cytokines and age shows that patients with COVID-19 with different outcomes are separate in the validation set. **(E)** The nomogram of the signature. **(F)** Calibration curve plots of the signature. The lowess fits the reference line well, showing that the signature is effective. **(G)** The decision curve analysis (DCA) of the signature in the training set. **(H)** The DCA of the signature in the validation set. Both decision curve analyses revealed that patients with COVID-19 can benefit from applying the LR signature.

The AUC of the model in the training set was 0.919 and in the validation set was 0.7236 (Figures 2A,B). Both were >0.7, which means that the model possessed good diagnosability. Besides, we found that the different groups were separated based on the hub variables (Figures 2C,D) in the PCA, indicating that these four variables might represent essential differences between different groups (Figure 2E). We drew a nomogram to apply our model in clinical work better. For evaluation, we performed calibration curve plots that fit well with the diagonal reference line (Figure 2F), indicating our model's great performance. DCA is a powerful method for assessing the degree of patient benefits. This research applied the DCA for the model in training and validation sets (Figures 2G,H). The DCA curves revealed that patients with COVID-19 could obtain net benefits through the logistic regression model. To establish a more comprehensive prognosis evaluation system, we applied Cox regression in Supplementary material 1.

### Establishing an ML model to classify patients and evaluate the model

**Figure 3:**
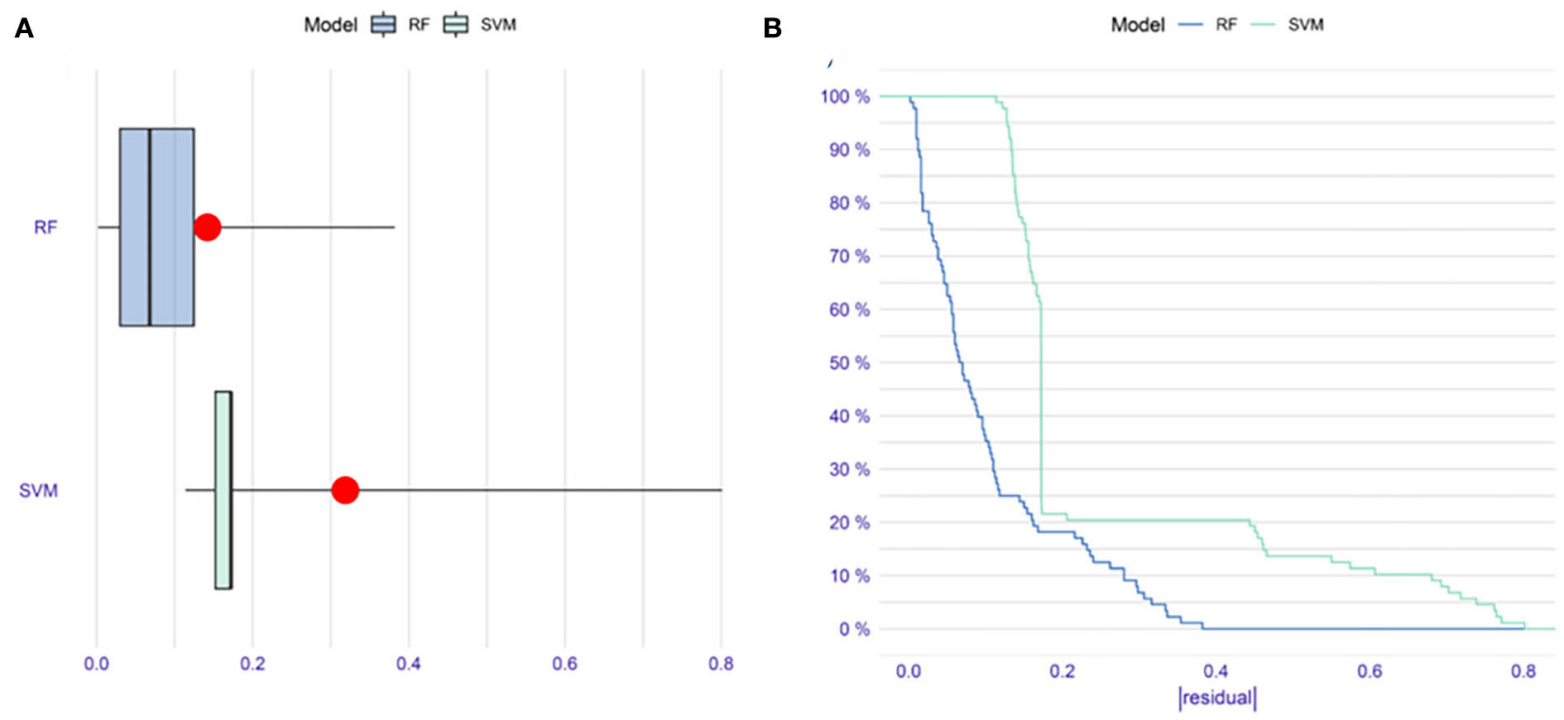
The comparison of random forest (RF) and support vector machine (SVM) methods in constructing models. **(A)** Boxplots of |residual|. The red dot stands for the root mean square of residuals. **(B)** Reverse cumulative distribution of |residual|. These results indicate that RF might be more suitable than SVM to construct a model in our research.

To improve the diagnostic performance of the model, we applied ML algorithms, including RF and SVM, to construct a new model. To reduce the subsequent unnecessary workload, first, we evaluated the residuals of the SVM and RF (Figures 3A,B). The results indicated that RF performed better with fewer residuals. Therefore, we decided to choose RF as the main ML algorithm to construct a model.

**Figure 4:**
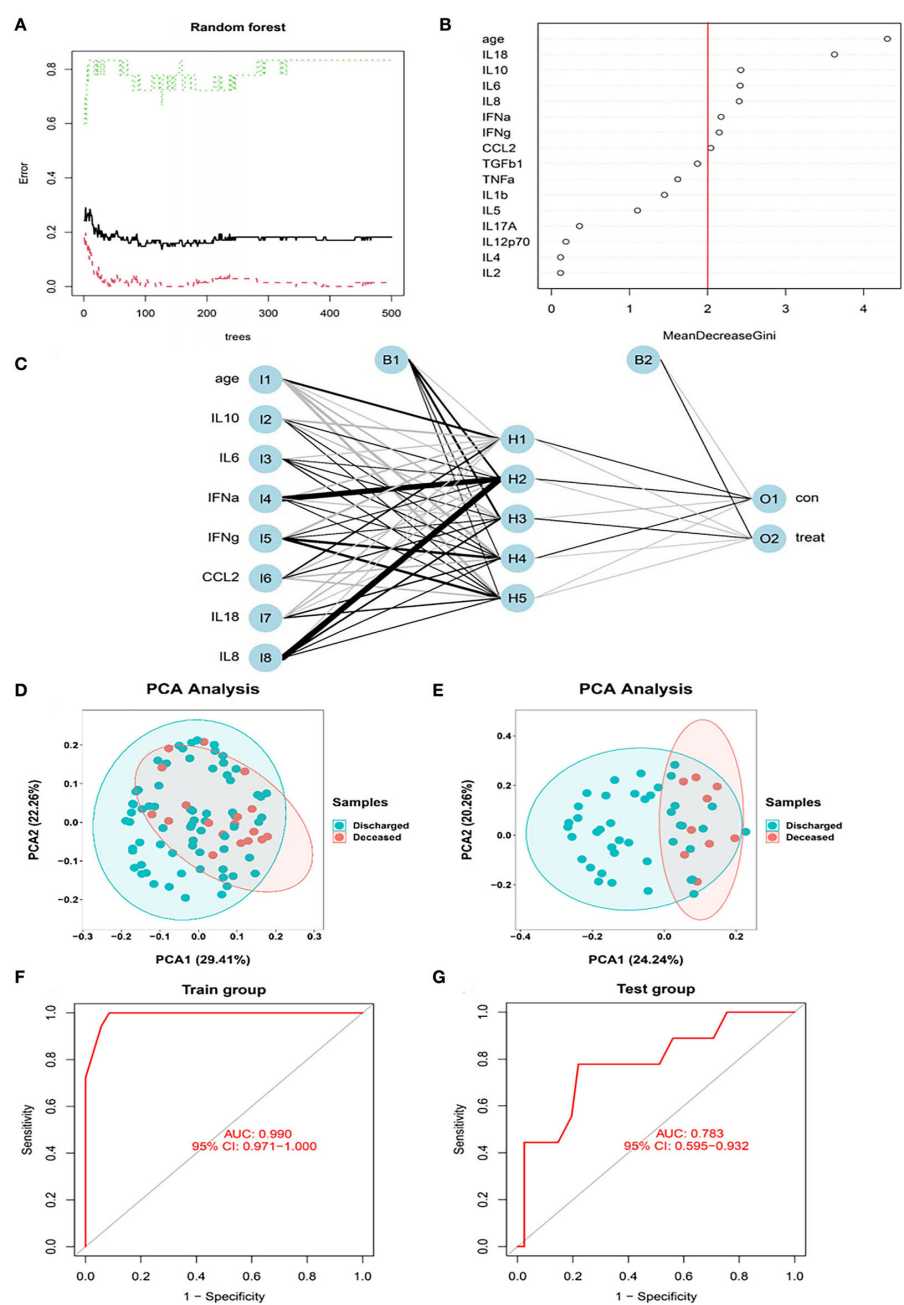
The RF modeling processes. **(A)** The influence of the number of decision trees on the error rate. After multiple repetitive operations, the error becomes stable gradually. **(B)** Results of the Gini coefficient method in the random forest classifier. We set importance=2 as a cutoff. **(C)** Neural network topology of the microarray with 8 input layers, 5 hidden layers, and 2 output layers. **(D)** The PCA of hub cytokines and age shows that patients with COVID-19 with different outcomes are separate in the training set. **(E)** The PCA of hub cytokines and age shows that patients with COVID-19 with different outcomes are separate in the validation set. **(F)** The ROC of signature in the training set. AUC is 0.99, indicating that the signature is perfect in the training set. **(G)** The ROC of signature in the validation set. AUC is 0.783, indicating that the signature is good in the validation set.

In the next step, all the variables (including cytokines, sex, and age) were entered into the RF classifier. We set the optimal parameter mtry to 2 as a default setting. The optimal number of trees in the classifier was set as 500, maintaining a low error for the classifier (Figure 4A). We used the perspective of reducing mean square error to measure the variable importance of the results (Gini coefficient). To keep our model succinct, we identified hub variables using a cutoff of importance >2 (Figure 4B). After obtaining the hub variables, we constructed an ANN model through the R package “neuralnet”. Two parallel training processes were used to construct a scoring model based on the training set. The ANN topology of the training set contained eight input layers, five hidden layers, and two output layers (Figure 4C).

The PCA of hub cytokines from RF and age shows that patients with COVID-19 with different outcomes are separate in both training and validation sets, revealing that the model possesses good discrimination (Figures 4D,E). In the training set, the AUC of the model was 0.99 (Figure 4F). Additionally, in the validation set, the AUC came to 0.783 (Figure 4G). The results indicated that the model we built possessed advantages in some situations.

### Constructing a DT to classify patients and evaluate the model

**Figure 5:**
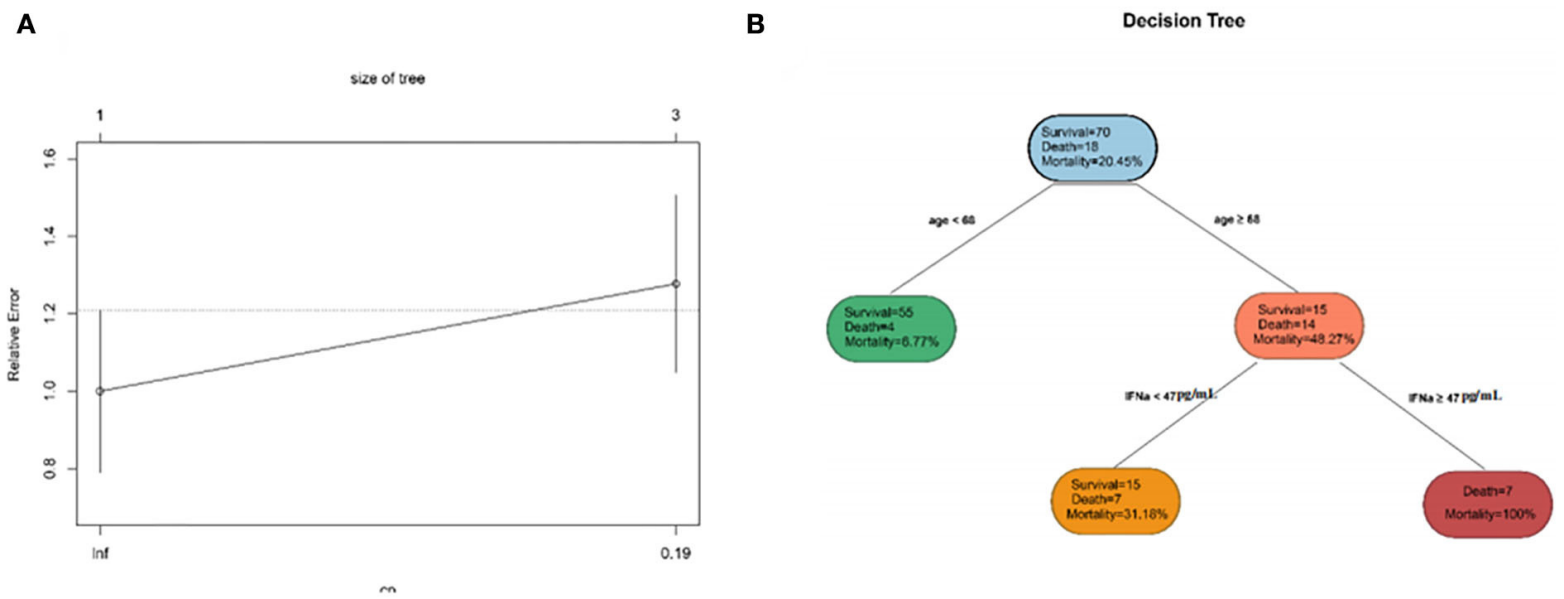
The characteristics of decision tree (DT). **(A)** The relative errors of the DT model and tree size when the complexity parameter comes to an ideal value. **(B)** The inter-relationship among selected clinical indicators.

To simplify the model and improve the feasibility of models for clinical application, we performed DT for all the variables previously mentioned. DT showed the interrelationship among the selected variables screened by the DT algorithm. The DT became the most straightforward tree when the complexity parameter (CP) was 0.1944444 (Figure 5). The first filtration age was (≥68 years), and the mortality was 48.27%. The second combination was IFN-α≥47 pg/ml. The mortality for patients with COVID-19 within the double combination was 100%. In the training set and validation set, the accuracy of the DT model was 0.875 and 0.86, respectively. The results revealed that the DT model considered both effectiveness and concise usability.

### Models' performance evaluation

**Figure 6:**
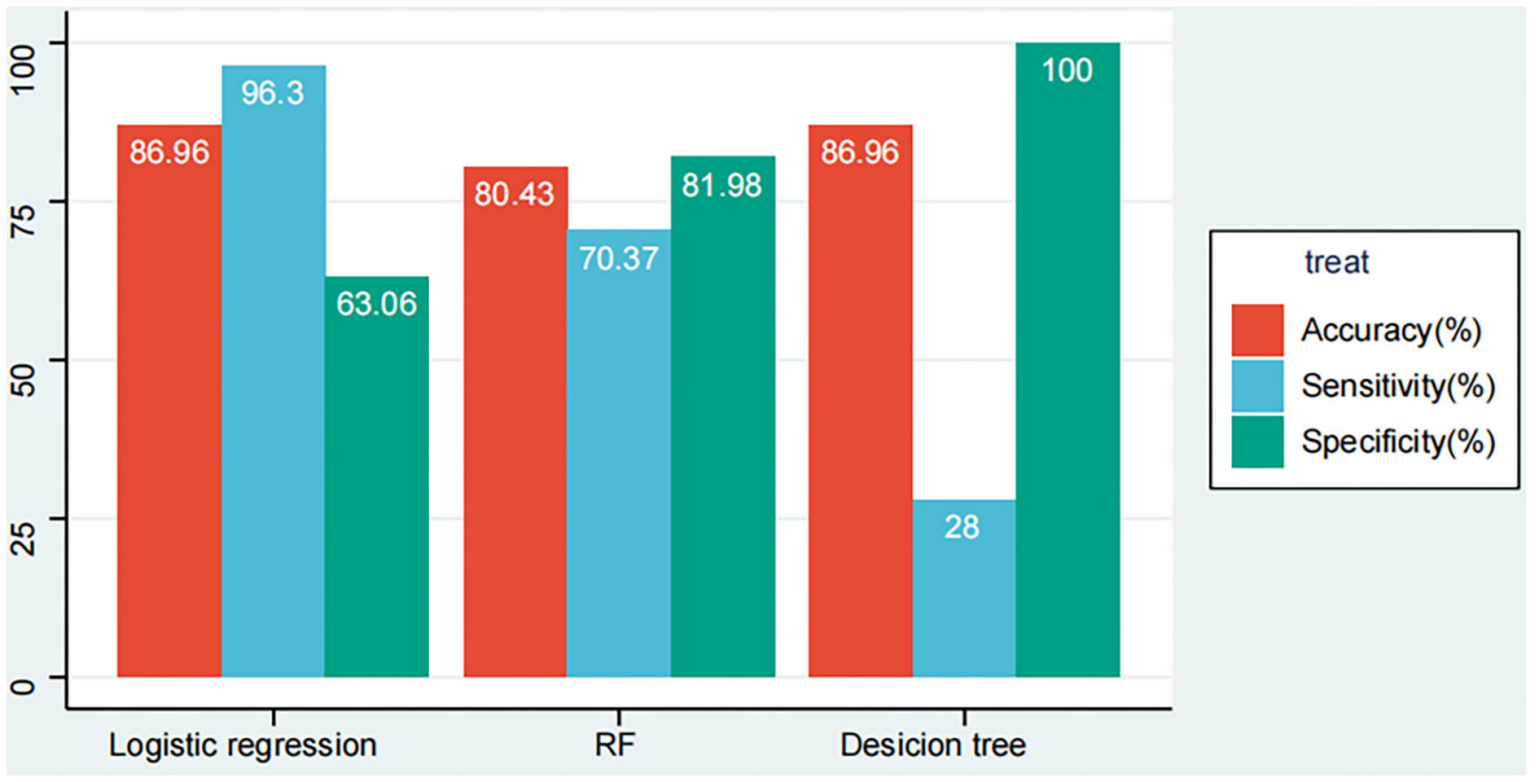
LR, RF, and DT models' performance evaluation results in the whole dataset.

We listed the results of the three models' performance in overall patients with COVID-19 in our cohort (the data from the training set and the validation set are merged, 138 patients). According to Figure 6, the models developed by logistic regression happened to be the highest in accuracy with 86.96% when compared with other models developed by the RF model and the DT model have 80.43 and 85.51%. While for sensitivity that shows the mortality rate of patients with COVID-19 correctly by the models, the logistic regression model seems to be the beat one with 96.3%, followed by the RF model with 70.37% and the DT model with 25.93%. Additionally, for specificity showing the survival rate of patients with COVID-19 correctly by the models, the DT model emerged to be the best one with 100%, followed by the RF model with 81.98% and the logistic regression model with 63.06%.

## Discussion

This research found that old age and several inflammatory cytokines played a crucial role in promoting severe COVID-19. We constructed multiple predictive prognostic models based on these factors to identify patients with COVID-19 at high risk of death at hospital entry. The models were validated using external datasets, and all models' performance is satisfactory. What we did may provide a novel insight into evaluating COVID-19 patients' conditions.

SARS-CoV-2, caused by COVID-19, spread worldwide at an unpredicted speed and brought profound and unfolding impacts on every aspect of human life. Previous studies revealed that patients with COVID-19 expressed huge heterogeneous characteristics, ranging from asymptomatic to losing lives (14, 15). COVID-19 infection affects various systems in the human body, including the immune system, leading to changes in the patient's internal environment and inflammation. Gao et al. (16) reported that pro-inflammatory cytokines were highly associated with severe disease. Previous studies had found that several cytokines were closely related to the development of COVID-19 but simply considered individual cytokines as predictive indicators (17, 18). COVID-19 causes immune dysregulation accompanied by multiple cytokine disturbances, which is hard to evaluate scientifically with one variable. Therefore, an effective inflammatory cytokines signature to comprehensively evaluate the COVID-19 patient's immune status must contain multiple variables. Inflammatory cytokines are texted in serum, which is easy to obtain from inpatients. The 12 inflammatory cytokines, the most essential and common series of cytokines, are sufficient to evaluate the patient's immune status.

In the research, we filtered out some prominent inflammatory cytokines in predicting the prognosis of COVID-19. IFN-α also has immunoregulatory effects, which might activate inflammatory responses and cause uncontrolled pathogenic damage (19). Krämer et al. (20) indicated preferential IFN-α responses in severe COVID-19 and declared that IFN-α was associated with a poorer COVID-19 infection outcome. Our research found that early high IFN-α signatures were hazardous features of poor prognoses for patients with COVID-19. Patients with COVID-19 with old age and elevated IFN-α levels suffered a very extreme risk of death. Systemic and autocrine IL-8 loops were essential neutrophil activation factors for immunopathology, triggering multiple cell dysfunctions (21). A previous study indicated that in patients with severe COVID-19, IL-8 might be a prognostic indicator for in-hospital death and a target for an effective treatment strategy (22). IL-8 was included in both the logistic regression and RF models in our research, which declared a crucial clinical value for this inflammatory cytokine. IL-6 is one of the most prominent inflammatory cytokines. According to Mojtabavi et al. (23), the elevated IL-6 level was an independent risk factor for adverse COVID-19 outcomes. There had already been several treatment strategies based on IL-6. Some results of them were reported as encouraging (24, 25). In our study, IL-6 significantly differed between death and survival cases in training and verification sets. This result revealed that IL-6 was a stable prognostic factor that could be applied on a large scale. Maione et al. (26) indicated IL-17A as a silent amplifier of cytokine storm in patients with COVID-19, activating several inflammatory pathways. In the clinical work, researchers identified IL-17A as a target to develop therapeutic strategies and made some progress (27). In this study, we screened out a possible link between elevated IL-17A levels and COVID-19 mortality.

Due to our work, we found that age was a key factor for all models and scored the highest in the RF model. There exist significant differences in age between survival and death cases. O'Driscoll et al. (28) declared that patients with COVID-19 aged older than 65 years suffered from higher mortality. Chen et al. (29) stated that age was related to declining and dysregulation of immune function, which heightened vulnerability to COVID-19 in elders. Previous researchers set the age>65 years as a high risk for severe COVID-19 outcomes (28, 30). Our DT sets age>68 years as a cutoff of the first combination, similar to those in previous reports. These results indicated that age played a major role in contributing to COVID-19 mortality.

Considering all models involved in this study comprehensively, the models developed with RF and logistic regression happened to be two well-rounded models, with considerable AUC in the training set and AUC = 0.783 in the validation set and high accuracy. However, the complexity of these models might bring some inconvenience in clinical applications. The model constructed by DT had good accuracy, specificity, and convenience. As for areas with underdeveloped medical conditions, it is an excellent choice to apply the DT model we constructed which was a simple model with good accuracy.

Building COVID-19 prognostic models through blood inflammatory cytokines levels is a novel thought. Thus, our research suggests that more extensive cohort studies should be conducted to reveal the role of inflammatory cytokines in predicting long-term post-COVID-19 complications.

## Conclusion

In this research, we identified that advanced age, IFN-α, IL-8, and IL-6 have been identified as potential prognostic predictors of COVID-19 outcomes by multiple models in our research, which indicated that these cytokines might play a vital role in the progression of SARS-CoV-2. Therefore, we advised that accurate and quantitative detection of the inflammatory cytokines could be performed when necessary. The RF, logistic regression, and DT models based on blood inflammatory cytokines performed well in identifying patients with COVID-19 at risk of death. We strongly suggest that the models developed with RF and logistic regression should be applied in the regions with more abundant medical resources, and the model developed by DT could be used in the regions with less abundant medical. The models we developed can assist doctors in applying individual strategies to different risk cohorts to perform early intervention and treatment to benefit patients with COVID-19.

## Data availability statement

The data and codes we used in this passage could be obtained from the corresponding author with reasonable requests. Requests to access these datasets should be directed to SS, sunshiren@medmail.com.cn.

## Ethics statement

Ethical review and approval was not required for the study on human participants in accordance with the local legislation and institutional requirements. Written informed consent for participation was not required for this study in accordance with the national legislation and the institutional requirements.

## Author contributions

ZY and XL conducted the analysis and prepared an initial draft. JZ collected data. SS offered ideas for the project. All authors contributed to the article and approved the submitted version.

## Funding

This study was funded by the Special Scientific Research Project of Shanghai Medical Assistance Team from Air Force Military Medical University in 2022, 2022YH-1009.

## Conflict of interest

The authors declare that the research was conducted in the absence of any commercial or financial relationships that could be construed as a potential conflict of interest.

## Publisher's note

All claims expressed in this article are solely those of the authors and do not necessarily represent those of their affiliated organizations, or those of the publisher, the editors and the reviewers. Any product that may be evaluated in this article, or claim that may be made by its manufacturer, is not guaranteed or endorsed by the publisher.
