# Supplementary material for: Identification of hospitalized mortality of patients with COVID-19 by machine learning models based on blood inflammatory cytokines
Source: Front Public Health. 2022 Nov 17;10:1001340. doi: 10.3389/fpubh.2022.1001340 (PMC9715399; doi:10.3389/fpubh.2022.1001340)
Supplement: Supplementary file 1 [file Data_Sheet_1.PDF]

Supplementary Material

Building a Cox regression model to classify patients and evaluate the model

Mueller et al. did not directly report the number of days before the events which caused the specific time before the events to be unknown. So we added "Days from symptom onset" and "total_days_in_hospital" to roughly estimate the days before the events. As shown in supplementary figure 1A, all the patients in the training set got their final events before 90 days. Multiple Cox regression was performed in the training set to construct a model. Sex age, TGFb1, IFNg, IFNa, IL18 and IL12p70 were identified as the hub factors associated with the prognosis of COVID-19 patients (Supplementary table1). We evaluated the model with ROC in the training and testing sets (Supplementary figure 1B-C). The AUC of the training and testing sets were 0.7908 and 0.6404, respectively. The Cox model might not perform better than the logistic regression model in the diagnosis due to the low AUC. For further research, we used the Cox model's DCA in both data sets (Supplementary figure 1D). However, the DCA in the training set showed that the Cox model's application could not benefit patients. What is more, this model consisted more inflammatory cytokines than logistic regression model. These results all showed that the Cox model has no clinical value which revealed that Cox regression might not be a good choice for our research.

Supplementary table 1. The results of multiple Cox analysis in the training set

Variables	_t	Coef.	HR	Std. Err.	z	P>z	95% Conf		Interval
TGFb1	-0.0000555	0.999944502	2.54E-05	-2.18	0.029	-0.00011	0.999895	-5.65E-06	0.999994
IFNg	-0.2685444	0.764491479	0.080616	-3.33	0.001	-0.42655	0.652758	-0.1105397	0.895351
IFNa	0.0183546	1.018524081	0.008291	2.21	0.027	0.002104	1.002106	0.0346053	1.035211
IL18	0.0031069	1.003111731	0.001053	2.95	0.003	0.001044	1.001044	0.0051699	1.005183
IL12p70	1.397094	4.043432662	0.705941	1.98	0.048	0.013476	1.013567	2.780712	16.1305
Sex	-2.210172	0.109681782	0.706423	-3.13	0.002	-3.59474	0.027468	-0.8256086	0.437968

Supplementary figure 1 The evaluation of the Cox model.

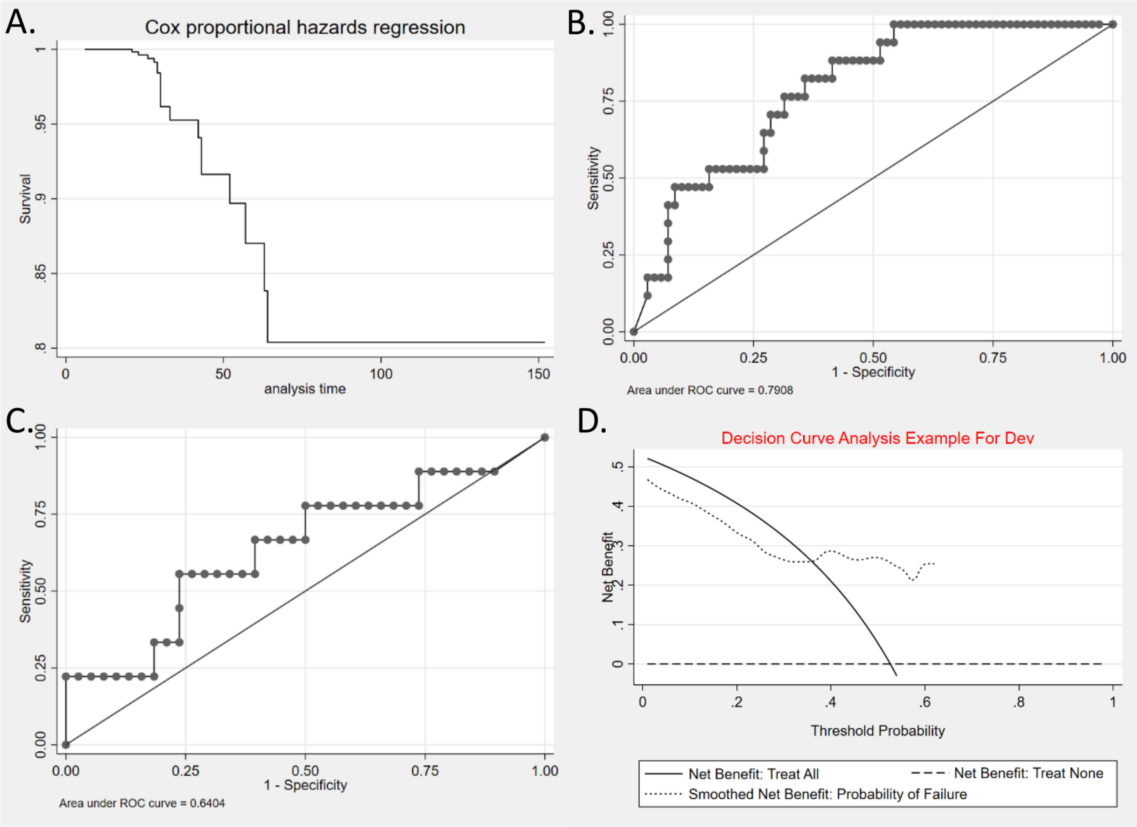

(A) The survival rate of the COVID-19 patients in the training set. (B) The ROC of the Cox model in the training set. (C) The ROC of the Cox model in the validation set. (D) The DCA of the Cox model in the training set.
